# Systematic Review of Prevalence Studies of Progressive Supranuclear Palsy and Corticobasal Syndrome

**DOI:** 10.1002/mdc3.13489

**Published:** 2022-06-28

**Authors:** Diane M.A. Swallow, Cindy S. Zheng, Carl E. Counsell

**Affiliations:** ^1^ Institute of Applied Health Sciences University of Aberdeen Aberdeen; ^2^ University of Aberdeen Medical School University of Aberdeen Aberdeen United Kingdom

**Keywords:** prevalence, progressive supranuclear palsy, corticobasal degeneration, systematic review

## Abstract

**Background:**

High‐quality prevalence studies are important in estimating the burden of disease in a population, thus informing priority setting, resource allocation, delivery, and use of health services.

**Objectives:**

This study was undertaken to systematically review the methods and results of previous prevalence studies of progressive supranuclear palsy (PSP) and corticobasal syndrome (CBS) and make recommendations for future studies.

**Methods:**

A total of 2 authors independently identified original articles that described the prevalence of PSP or CBS using several comprehensive and overlapping search strategies, assessed study quality, and extracted relevant data. Descriptive and pooled analyses were performed as appropriate.

**Results:**

A total of 16 studies were identified in PSP and 9 studies in CBS, with highly heterogeneous methods of case definition, identification, and verification in identified studies. Few studies were deemed of necessary quality or methodological homogeneity to justify a full meta‐analysis. In addition, few studies reported age‐ and sex‐stratified results. The best 3 prevalence studies in PSP gave a pooled rate of 7.1 per 100,000 per year, whereas the pooled rate in 2 CBS studies was roughly 3 times lower at 2.3 per 100,000 per year. Based on crude rates, there was little evidence to suggest clear sex differences in the prevalence of PSP or CBS or that the prevalence of PSP had increased over time, but some evidence to suggest that prevalence may increase with increasing age.

**Conclusion:**

Given the paucity of prevalence studies in PSP and CBS, further high‐quality prevalence studies are necessary.

Progressive supranuclear palsy (PSP) and corticobasal degeneration (CBD) are progressive neurodegenerative diseases characterized pathologically by accumulation of the tau protein.^1^ In CBD, as a greater diversity of clinical presentations in those with CBD pathology has been recognized, the classic clinical phenotype is now increasingly, and perhaps most accurately, known as corticobasal syndrome (CBS) with the term CBD increasingly restricted to pathologically confirmed cases. Most individuals with PSP and CBS quickly become dependent on others for care due to rapidly accruing motor and cognitive disability, with an estimated overall survival of 3 to 8 years.^1^, ^2^, ^3^

High‐quality prevalence studies are of particular importance in estimating the burden of disease in a population to inform priority setting, resource allocation, delivery, and use of health services. This article reviews the methods and results of previous prevalence studies of PSP/CBS, considers the challenges in conducting high‐quality prevalence studies in these conditions, and makes recommendations for the conduct of future prevalence studies in PSP/CBS.

## Methods

### Search Strategy

MEDLINE (Ovid), EMBASE (Ovid), Web of Science (Thompson Reuters), Latin American and Carribean Health Sciences Literature (LILACS) (Bireme), and CINAHL Database (EBSCO) were searched for relevant primary studies in PSP/CBS (detailed search strategies in Appendix S1). A total of 2 authors (D.M.A.S., C.S.Z.) independently screened all titles, abstracts, and full‐text articles to select studies for inclusion. Discrepancies were resolved through discussion, with any lack of consensus resolved by a third author (C.E.C.). The reference lists of all identified primary studies in PSP/CBS were searched.

As prevalence figures for PSP/CBS may be reported within studies undertaken to determine the prevalence of parkinsonism or frontotemporal dementia (FTD), searches were also performed for systematic reviews of prevalence studies of Parkinson's disease (PD), parkinsonism, and FTD so that the reference lists of identified reviews could be screened for relevant primary studies. Additional searches to identify prevalence studies beyond the years of interest in identified systematic reviews were also undertaken.

### Eligibility Criteria

Studies had to be full text (abstracts or conference proceedings were excluded as methodological quality could not be determined) and report an original study that provided a population‐based prevalence rate for PSP or CBS.

### Data Extraction and Analysis

A total of 2 authors (D.M.A.S., C.S.Z.) independently extracted the following information: first author, year of publication, study aims, country, recruitment period, prevalence day, population denominator size and source, case definition with inclusion and exclusion criteria, sources and methods of case identification and assessment (including the percentage of possible cases examined), response rates, and availability of follow‐up. The number of prevalent cases, population size or denominator, crude prevalence rates, age‐ and sex‐stratified prevalence rates, mean age at disease onset, and details of standardized populations were also extracted where available. Discrepancies in data extraction were resolved with a third author (C.E.C.) if required. Where not reported, prevalence rates were calculated when raw numbers were reported. The 95% confidence intervals (CIs) for the crude rates were calculated assuming a Poisson distribution.

The prevalence rates in different studies were included in a random‐effects meta‐analysis (DerSimonian and Laird model) only if sufficiently similar methods justified such comparisons. For this purpose, we decided that studies should have reported population prevalence rates unrestricted by age, had a reliable population denominator source, used similar diagnostic and exclusion criteria for case definition, included more than 1 method of case ascertainment to minimize the number of cases missed, and attempted to verify the diagnosis by either clinical examination or medical record review. Statistical analyses were performed using Stata version 15 (StataCorp LP, College Station, TX).

## Results

### Search Results

**FIG. 1:**
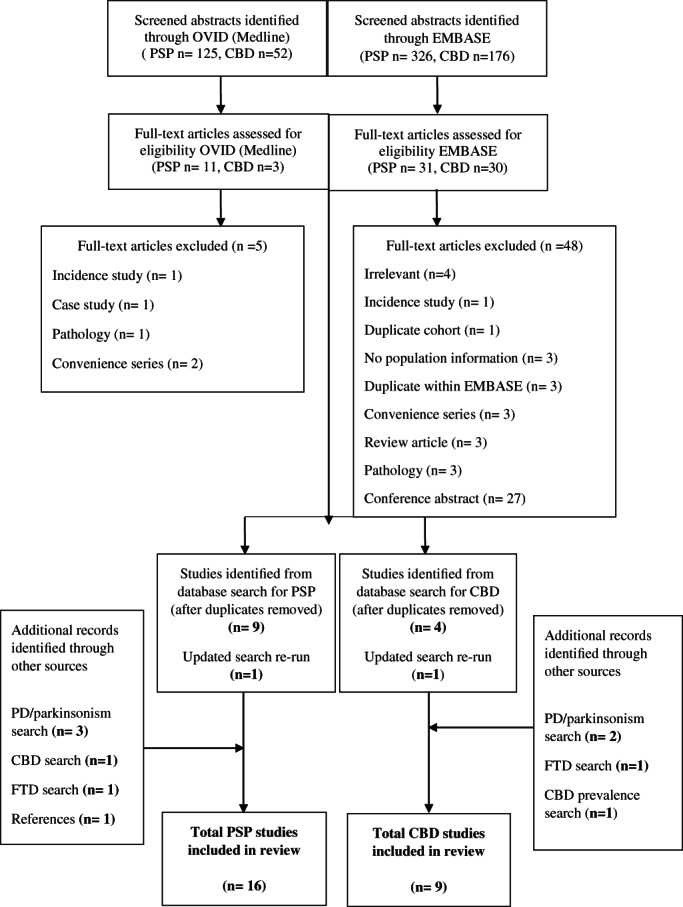
Flow diagram showing search strategies and sources of included prevalence studies in progressive supranuclear palsy and corticobasal degeneration. CBD, corticobasal degeneration; FTD, frontotemporal dementia; PD, Parkinson's disease; PSP, progressive supranuclear palsy.

In total, 16 studies were identified for PSP and 9 studies for CBS (Fig. 1). No unique references were identified through Web of Science, LILACS, or CINAHL Database searches.

### Study Methodology

Detailed summaries of the methods of the included studies are shown in Tables S1 and S2.

#### Study Purpose

A total of 5 studies^4^, ^5^, ^6^, ^7^, ^8^ were conducted specifically to determine the prevalence of PSP, and none were conducted to determine the prevalence of CBS. Of the studies, 14 identified PSP/CBS as part of broader studies of PD, parkinsonism, or dementia.

#### Methods Used to Define the Population Denominator

The majority of studies used official census estimates,^6^, ^8^, ^9^, ^10^, ^11^, ^12^, ^13^, ^14^, ^15^ regional registry/city office statistics^4^, ^12^, ^16^, ^17^, ^18^, ^19^ or numbers registered with general practices^6^, ^7^ to establish their population denominator. A total of 2 studies^20^, ^21^ from the same area used lists kept by 3 community public health nurses; 1 study^22^ used a preexisting study cohort, and in 1^5^ the population denominator source was not stated. In 4 studies, the target study population was sampled using random and nonrandom techniques.^7^, ^13^, ^14^, ^18^

#### Methods of Case Definition

Diagnostic criteria were the most commonly used method of standardizing case definition. In PSP, the majority of studies^4^, ^5^, ^6^, ^7^, ^9^, ^12^, ^20^, ^22^ used the National Institute of Neurological Disorders and Stroke– Society for PSP, Inc. (NINDS‐SPSP) 1996 international consensus criteria to diagnose PSP,^23^ 5 of which categorized diagnoses as probable or possible.^4^, ^5^, ^6^, ^7^, ^9^ One study referenced the NINDS‐SPSP criteria without explicitly stating its use.^16^ In another study,^10^ the Neuroprotection and Natural History in Parkinson Plus Syndromes (NNIPPS) study criteria was used.^24^ A total of 2 studies^8^, ^18^ conducted prior to the NINDS‐SPSP criteria used alternative diagnostic criteria: in 1 study, the authors formulated their own diagnostic criteria for PSP^19^; in the other,^18^ the diagnosis of PSP was based on clinical criteria developed from a pathological series of PSP cases. Only 1 study,^4^ conducted prior to the recent publication of the International Parkinson and Movement Disorder Society (MDS) PSP subtype criteria,^25^ attempted to determine the prevalence of PSP subtypes according to published descriptions.^1^ In 4 studies, diagnostic criteria were not stated.^11^, ^17^, ^19^, ^21^

Only 2 identified studies^10^, ^12^ defined their cases as CBS, both of which used the 2013 Armstrong criteria^26^ (neither categorizing their cases as probable or possible). The remainder of studies defined their cases as CBD despite the lack of pathological confirmation. A total of 2 studies^9^, ^14^ used the Kumar criteria,^27^ and 1 study^16^ used Lang's criteria.^28^ Two studies^13^, ^15^ referenced *Diagnostic and Statistical Manual of Mental Disorders, Fourth Edition* criteria, and in 1 study the diagnostic criteria were not stated.^11^

A total of 8 studies restricted their case identification and population denominator population by age, reporting only age‐stratified rates: 30 to 64 years,^11^ <65 years,^15^ ≥50 years,^14^ ≥55 years,^18^, ^19^ >60 years,^22^ and ≥65 years.^20^, ^21^ The size of the target population in studies unrestricted by age ranged from 1,742^22^ to 59,236,500.^6^

#### Methods to Identify Possible Cases

Age‐restricted, door‐to‐door initial phase questionnaire or cognitive screening was used in 4 studies.^18^, ^19^, ^20^, ^21^ In 4 studies in which sampling was employed,^13^, ^14^, ^18^, ^21^ contact with all members of the sampled population was attempted. In 2 community studies, general practices were sampled, and all registered patients screened.^6^, ^7^ In 1 study, all individuals participating in a prospective study on aging were screened.^22^ One study^4^ used a single source: annual medical record surveys conducted at a single university hospital. In 2 studies^8^, ^9^ identification of cases was by referral only but across multiple sites.

Most of the remaining studies used multiple sources of case identification, including direct health care professional referral (regional specialist clinics,^10^ multiple medical specialities,^6^, ^9^, ^11^, ^15^ neurologists only,^5^, ^8^ primary care clinicians,^11^, ^15^, ^16^, ^17^ allied health professionals/specialist nurses^10^, ^11^, ^15^) and patient self‐referral.^8^, ^10^, ^16^ Nearly all studies seeking direct referrals issued referral reminders^8^, ^9^, ^10^, ^11^, ^17^ or surveyed more than once.^5^ Patient consent for case notification was required in 1 study^10^ and for screening in another study.^19^ Hospital records or databases were used in 10 studies to identify cases. In 4 studies,^6^, ^11^, ^12^, ^15^ inpatient *International Classification of Diseases, Tenth Revision* (*ICD‐10*) diagnostic codes were searched. In 2 studies,^6^, ^7^ keyword searches of diagnostic and therapeutic primary care patient databases were performed. The specificity of diagnoses sought varied depending on the primary purpose of the study. Pharmacy records were screened in 4 studies for antiparkinsonian medication use.^6^, ^7^, ^16^, ^17^ Community nursing homes were targeted in 4 studies^5^, ^8^, ^12^, ^17^ A total of 3 studies^8^, ^12^, ^17^ contacted all nursing homes in the population of interest to seek referrals^8^, ^16^ or screen nursing records.^12^ In the fourth study,^5^ screening examinations were undertaken in 10 registered nursing homes, although what proportion of residents in this population this represented is unclear. Additional methods of case identification included referrals via patient charities,^10^ national surveillance units,^6^ clinical research networks,^10^ national mortality data,^6^ and insurance systems.^9^

The duration of case identification was variably stated. A total of 2 studies^4^, ^5^ undertook multiple, minimally annual, cross‐sectional surveys. Several studies explicitly stated the duration or extent of their identification period.^8^, ^10^, ^11^, ^12^, ^13^, ^14^, ^18^, ^19^, ^21^, ^22^ Some studies included no information on the duration of their identification period^7^, ^9^, ^15^, ^20^ or did not provide this information for all of their methods of case identification.^6^, ^16^, ^17^ Very few studies explicitly evaluated the success of their methods of case identification or response rates^6^, ^11^, ^12^ or considered the representativeness of cases.^6^, ^7^, ^14^

#### Methods to Verify Included Cases

A total of 11 studies attempted to verify cases by neurologist or movement disorder specialist examination of all identified cases^4^, ^6^, ^8^, ^10^, ^13^, ^14^, ^16^, ^17^, ^18^, ^19^, ^22^ or after an initial medical records screen,^9^ cognitive screen,^20^, ^21^ or keyword or pharmacy review.^6^, ^7^, ^19^ In 2 studies^11^, ^12^ and in the national prevalence component of a third study,^6^ case verification was by case note review alone. In 1 study, verification was dependent on the source of the identified case.^5^ The proportion of patients examined across studies ranged from 47%^15^ to 100%,^20^ but this was not always stated or clear.^4^, ^5^, ^13^, ^21^ A number of studies made multiple efforts to contact patients to examine them.^6^, ^7^, ^16^, ^18^

The extent of cognitive impairment also influenced case inclusion. In 1 study,^16^ classification of those with atypical parkinsonism was not attempted when severe dementia was present. In an earlier study by the same authors,^17^ those with severe dementia were excluded.

#### Prospective Follow‐Up

Only 3 studies^6^, ^7^, ^18^ employed follow‐up to clarify the diagnosis. In 1 study,^18^ 78% of individuals underwent at least 1 further neurological examination. In another study,^6^ if medical records indicated suggestive features but without a definite diagnosis, a further 6 months of correspondence was reviewed to identify emerging diagnoses. In the third study,^7^ patients with a diagnosis of parkinsonism, PSP, or multiple system atrophy completed regular questionnaires about the development of atypical features and symptoms of progression. Patients with no definitive diagnosis or with atypical features at the first visit or during follow‐up were reassessed.

#### Other Methodological Issues

The prevalence day was stated in all but 3^15^, ^19^, ^22^ studies. However, it was not always explicitly stated that all identified cases were alive and residing in the area on the prevalence day.

### Prevalence Rates

#### Progressive Supranuclear Palsy

**FIG. 2:**
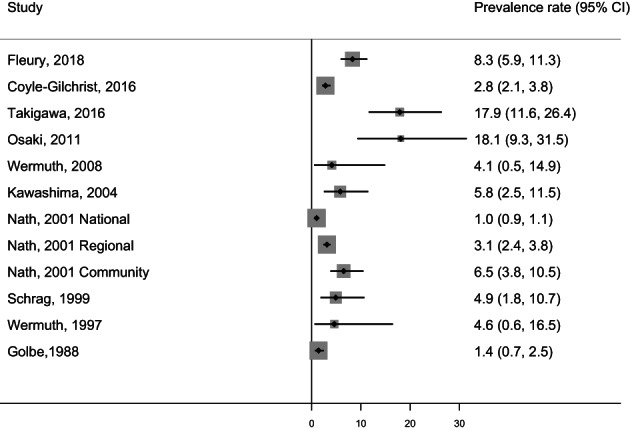
Prevalence rates per 100,000 (unrestricted by age) in progressive supranuclear palsy. CI, confidence interval.

The population/patient numbers and age‐ and sex‐stratified crude prevalence rates per 100,000 in the included studies on PSP are shown in Supplementary Table 3. Crude overall prevalence rates in studies unrestricted by age are summarized in Figure 2. In studies unrestricted by age, crude prevalence rates ranged from 1.0 per 100,000 (95% CI: 0.9, 1.1)^6^ to 18.1 per 100,000 (95% CI: 9.3, 31.5).^9^ No studies reported age‐ and sex‐stratified results.

Only 1 study^4^ determined the prevalence of individual PSP subtypes according to available descriptions.^1^ Of 25 patients, 20 (80%) were thought to have PSP–Richardson's syndrome (PSP‐RS), 3 (12%) PSP–parkinsonism (PSP‐P), and 2 (8%) PSP–pure akinesia with gait freezing (PSP‐PAGF), giving crude prevalence rates of 14.3 (95% CI: 8.7, 22.1), 2.1 (95% CI: 0.4, 6.3), and 1.4 (95% CI: 0.2, 5.2) per 100,000 for PSP‐RS, PSP‐P, and PSP‐PAGF, respectively. No study determining the prevalence of PSP has been reported since the recent publication of diagnostic criteria for PSP subtypes.^25^

With regard to geographical variation, 3 prevalence studies were conducted in England, all of which reported similar results: 6.5 and 4.9 per 100,000 in smaller community‐level studies^6^, ^7^ and slightly lower rates (3.1 and 2.8 per 100,000) in larger regional studies with less detailed ascertainment.^6^, ^10^ A total of 3 studies (unrestricted by age) were conducted in Japan, 2 of which reported similar prevalence rates^4^, ^9^: 17.9 and 18.0 per 100,000, considerably higher than those described in all other prevalence studies in this review. Where only probable diagnoses are considered in these 2 studies, however, CIs overlapped with other identified studies reporting lower point estimates. The third Japanese study,^5^ conducted in the same geographical area in 1 of the aforementioned studies^4^ 10 years earlier, reported a prevalence rate of 5.8 per 100 000, similar to those reported by studies conducted in England.

With regard to possible time trends, 2 studies were conducted in Yonago City, Japan, 10 years apart. The later study^4^ showed a significantly higher age‐ and sex‐adjusted prevalence rate than the earlier study^5^: 11.9 versus 5.8 per 100,000. A total of 2 studies were also conducted in the Faroe Islands, again 10 years apart, but showed no change over time (4.6 vs. 4.1 per 100,000^12^, ^21^). Although conducted in different regions, 2 regional studies in England conducted 15 years apart also reported similar prevalence rates (3.1 vs. 2.8 per 100,000^3^, ^10^).

#### Corticobasal Syndrome

**FIG. 3:**
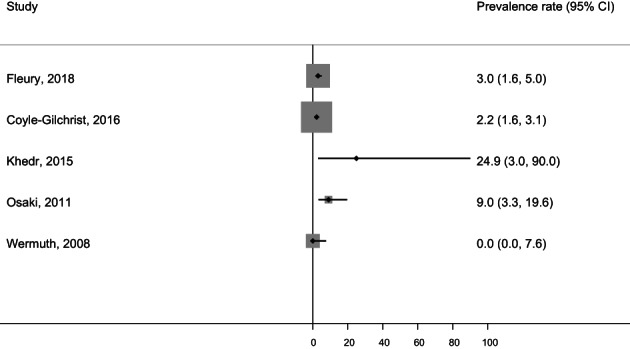
Prevalence rates per 100,000 (unrestricted by age) in corticobasal degeneration. CI, confidence interval.

Table S4 and Figure 3 show the population/patient numbers and age‐ and sex‐stratified crude prevalence rates per 100,000 in the included studies plus the crude overall prevalence rates in studies unrestricted by age. In studies unrestricted by age, the crude prevalence rates of CBS ranged from 0.0 per 100,000 (95% CI: 0.0, 7.6)^16^ to 25.0 per 100,000 (95% CI: 0.0, 59.0).^13^ No studies reported age‐ and sex‐stratified results.

#### Pooled Prevalence Rates

**FIG. 4:**
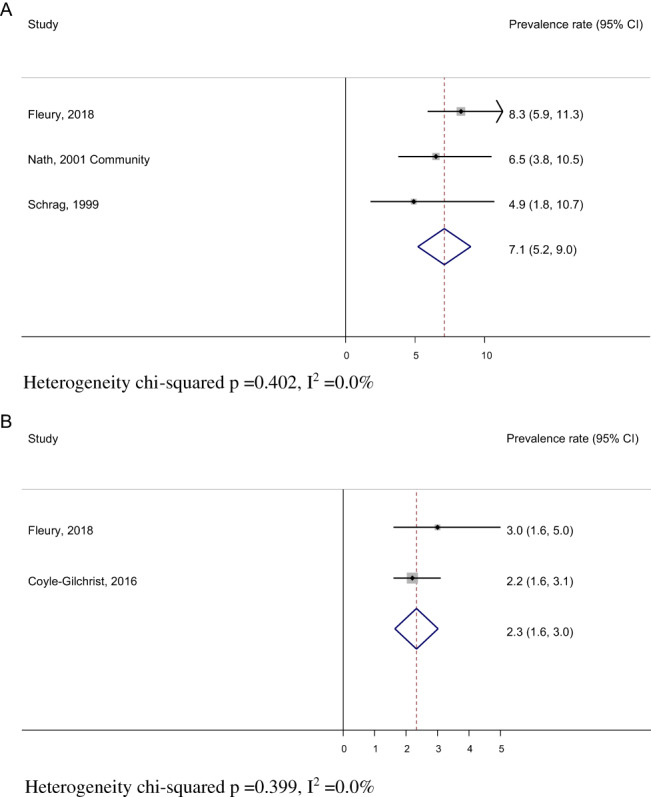
Pooled prevalence rate per 100,000 (unrestricted by age) in progressive supranuclear palsy (**A**) and corticobasal degeneration (**B**). CI, confidence interval.

Of 16 studies in PSP included for review, 3 studies^6^, ^7^, ^12^ were deemed of sufficiently similar methodology to pool individual prevalence rates, giving a pooled prevalence rate of 7.1 per 100,000 (95% CI: 5.2, 9.0) (Fig. 4A). Of 9 studies identified in CBS, 2 studies^6^, ^7^, ^10^, ^12^ had sufficiently similar methodology to pool prevalence rates (Fig. 4B), giving a pooled prevalence rate of 2.3 per 100,000 (95% CI: 1.6, 3.0).

## Discussion

### Strengths and Limitations

To our knowledge, this is the first systematic review of prevalence studies in PSP and CBS. We used inclusive, overlapping search strategies to identify eligible studies and assessed the methodology and results of each of the identified studies thoroughly. The review highlights the importance of search strategies that encompass syndromic (parkinsonism, FTD) in addition to PSP/CBS disease‐specific searches in that nearly half of all eligible studies were identified from the latter searches.

Although our search strategy was rigorous, we did not search Chinese or Japanese databases due to translation and resource limitations. Our failure to use a single quality assessment tool to evaluate the quality of identified studies could also be criticized. Although the Standards of Reporting of Neurological Disorders (STROND) checklist is recommended for reporting, no single critical appraisal tool has been recommended for systematic reviews of prevalence studies.^29^ Rather than arbitrarily select 1 appraisal tool, we assessed methodological quality using a breadth of available epidemiological quality criteria. When deciding on eligibility criterion for inclusion in pooled analyses, our decision to include more than 1 method of case ascertainment is also somewhat limited in that methods of case ascertainment may still vary widely within this quantitative parameter. Finally, any inferences about time trends or geographical differences must be made cautiously because differences in prevalence may reflect differences in incidence and/or survival as well as methodology.

### Prevalence Rates

The best prevalence studies in PSP gave a pooled rate of 7.1 per 100,000 per year, whereas the rate in CBS was roughly 3 times lower at 2.3 per 100,000 per year. Few identified studies were deemed of necessary quality (at least of reporting), or homogeneity, to justify a full meta‐analysis. Few studies reported age‐ and sex‐stratified populations, preventing standardization to a single population to facilitate comparisons. Based on crude rates, there is little evidence to suggest clear sex differences in the prevalence of PSP/CBS or that prevalence has increased over time. There is, however, some evidence to suggest that the prevalence of PSP increases with age. The higher prevalence of PSP reported in 2 Japanese studies in this review is interesting in light of a previous study that, having explored the distribution of the τ H1/H2 haplotype in different global populations, concluded that the H2 haplotype is probably exclusively Caucasian in origin. A proposed implication of this finding is that non‐Caucasian populations may have an increased incidence of H1‐associated tauopathies because such populations would have nearly twice as many H1 homozygotes.^30^ As previously cautioned, however, inferences regarding geographical differences must be made cautiously as differences in prevalence may reflect differences in incidence and/or survival as well as methodology.

### Challenges and Recommendations

One of the main benefits of this review is to highlight the scarcity of existing high‐quality prevalence studies in PSP/CBS and the methodological challenges involved in conducting such studies. We subsequently discuss methodological quality criteria that may inform the design of future prevalence studies in PSP/CBS. Decisions relating to study design should be consistently and transparently reported using the STROND checklist.

#### Population of Interest

Given the lower expected prevalence of PSP/CBS, the balance between a population size sufficient to contain PSP/CBS cases against the resources required to identify all cases should be carefully considered. A large population denominator will produce precise prevalence estimates with narrow confidence limits but may miss cases. Smaller populations of interest, in contrast, will generate wider CIs but improve surveillance. In 1 study^6^ identified in this review, for example, the authors identified a 20‐fold variation in the crude prevalence rate of PSP dependent on the population size surveyed. Whether whole or sampled populations are used, the source of the total population size or sampling frame should be clearly stated and derived from an independent, reliable source such as a population census or register.

In addition to total population size, age‐ and sex‐stratified population counts should be reported so that, if not described, age‐ and sex‐stratified prevalence rates can be calculated, along with CIs, enabling an appraisal of precision, but more important, standardization and comparison between studies. Given the rarity of PSP/CBS in patients aged <55 years, we suggest age strata cutoffs of <55, 55 to 64, 65 to 74, 75 to 84, 85 to 94, and ≥95 years.

#### Case Ascertainment

In general, case ascertainment should maximize sensitivity and reduce the risk of under‐ascertainment. In a larger population, there is a greater likelihood of missing or misclassifying cases due to a greater reliance on passive case ascertainment.

Given the phenotypic variability and overlap in PSP/CBS, maximally sensitive initial inclusion criteria would ideally be used, such as searching for all parkinsonian or FTD syndromes, capturing all possible motor and cognitive predominant PSP/CBS disease subtypes with subsequent application of specific diagnostic criteria by experts. In several studies in this review, case ascertainment was restricted to motor predominant cases, failing to classify,^16^ or actively excluding^17^ those with severe dementia or individuals who developed dementia prior to their motor symptoms.^7^ Eligible motor phenotypes were also restricted. In 3 studies,^6^, ^7^, ^19^ cases were selected from individuals with evidence of parkinsonism. Consequently, cases presenting with falls but without other evidence of parkinsonism may have been missed. Conversely, in 2 age‐restricted studies reporting PSP prevalence rates,^20^, ^21^ cases were selected from those screening positive in an initial phase cognitive screen.

In general, the use of multiple overlapping sources of both active and passive case ascertainment is the best approach to ensure eligible cases are not missed. Cases identified should be individually identifiable so that cases from multiple sources can be reliably de‐duplicated to avoid overestimation of prevalence. Although in the United Kingdom most cases with PSP/CBS should be captured by targeting clinicians in secondary care, referrals from community general practitioners may be required. Due to rapidly accruing disability, nursing home screening should also be considered. The impact of this source of case identification on prevalence may be sizable; 1 community incidence study of parkinsonism, for example, found 33% of identified PSP/CBS cases were institutionalized at diagnosis/recruitment.^3^ This may have a differential impact on identification depending on the predominance of motor or cognitive features. Finally, patient self‐referral with subsequent confirmation of diagnoses with their responsible clinician could also be considered. Referrer nonresponse should be anticipated, and regular reminders sent.

Several studies in this review undertook medical record screening, a number of which used *ICD‐10* diagnostic codes. Searching unselected medical records would identify both diagnosed cases not referred by clinicians as well as those with suggestive clinical features pending a formal diagnosis. Searches using diagnostic coding may be limited by coding errors and the variety of potentially applicable codes, in particular for CBS that, unlike PSP, does not have a specific *ICD‐10* code. Text searching of clinic letters for suggestive clinical features (eg, backward falls), in addition to established diagnoses, could be important in PSP/CBS given their high rates of delayed diagnosis; however, this would be impossible in large populations, involve a significant time commitment, and be susceptible to error. Developments in natural language processing may improve the feasibility of this search methodology in the future.

An evolving research governance and regulatory landscape with increasing emphasis on data privacy may increasingly limit case notification and verification. Consent for case notification makes it near impossible to conduct rigorous prevalence studies in that inevitably consent will not be sought, be refused, or be impossible to obtain due to incapacity secondary to dementia, leading to under‐ascertainment. It may also limit the unselected review of outpatient correspondence or a search for nonspecific *ICD‐10* codes due to the perceived threats to patient confidentiality.

#### Case Definition

Variations in the applied diagnostic criteria could result in differences in prevalence, depending on the individual sensitivity or specificity of selected criteria. Overall, diagnostic criteria with the best validity should be used. The use of diagnostic consensus criteria where available would aid consistency and facilitate comparison between studies.

For future studies, the new MDS criteria for PSP would allow stratification by diagnostic certainty and subclassification of PSP subtype while also including cases other diagnostic criteria would exclude, for example, the NNIPPS criteria where the inclusion of rest tremor as an exclusion criterion could potentially exclude the PSP‐P subtype. Our pooled prevalence rate for this reason, based on the diagnostic criteria used, would perhaps be most accurately described as NINDS‐SPSP pooled prevalence. However, new criteria require further validation, and familiarization with and implementation of these relatively complex criteria in day‐to‐day clinical practice is likely to take time.

Although several diagnostic criteria have also been proposed for CBS, concordance between criteria is low. The more recent Armstrong criteria usefully include criteria for neuropathologically confirmed CBS subtypes as well as stratification by diagnostic certainty, but subsequent validation exercises have highlighted limitations in its specificity.^31^

#### Case Verification

Due to the high rates of misdiagnosis and delayed diagnosis in PSP/CBS, particularly in the earliest stages of the disease, an expert review of identified cases including follow‐up of diagnostically uncertain cases should be undertaken where possible. Ideally this would be based on clinical examination rather than case note review where relevant clinical details may be missing. If examination is impossible due to population size, other resource restrictions, patient frailty, or absence of consent, the source of the case referral should be considered and whether the duration of case identification was long enough to maximize case ascertainment and the diagnostic accuracy of emerging diagnoses. Stratification by diagnostic certainty, either probable or possible, should also be reported where possible.

While a definite diagnosis of PSP/CBD requires postmortem, extrapolation of prevalence estimates from existing postmortem series are likely to be misleading as such studies favor atypical or diagnostically uncertain cases. Until an adequately specific biomarker to facilitate a definite antemortem diagnosis in PSP/CBS is established, population prevalence studies would benefit from encouraging postmortem confirmation of diagnoses.

### Conclusion

For PSP, 3 rigorous, high‐quality studies^6^, ^7^, ^12^ were of sufficiently similar methodology to pool prevalence rates, giving a pooled prevalence rate of 7.1 per 100,000 (95% CI, 5.2, 9.0). For CBS, 2 sufficiently homogenous studies^10^, ^12^ were identified, giving a pooled prevalence rate of 2.3 per 100,000 (95% CI, 1.6, 3.0). Given the paucity of prevalence studies in PSP/CBS and the importance of such studies in informing health care and identifying time and geographical patterns, further high‐quality prevalence studies are necessary.

## Author Roles

(1) Research Project: A. Study Design, B. Data Acquisition, C. Interpretation and Analysis; (2) Manuscript: A. Writing of the First Draft, B. Review and Critique.

D.M.A.S.: 1A, 1B, 1C, 2A, 2B

C.S.Z.: 1B, 2B

C.E.C.: 1A, 1C, 2B

## Disclosures

**Ethical Compliance Statement:** The project was approved by the Public Benefit and Privacy Panel for Health and Social Care in Scotland and additional ethical committee approval or informed patient consent was not necessary for this work. We confirm that we have read the Journal's position on issues involved in ethical publication and affirm that this work is consistent with those guidelines.

**Funding Sources and Conflicts of Interest:** There are no conflicts of interest relevant to this work. Dr. Diane M.A. Swallow was funded by a clinical research fellowship jointly funded by the Chief Scientist Office of the Scottish Government and the PSP Association.

**Financial Disclosures for the Previous 12 Months:** The authors declare that there are no additional disclosures to report.

## Supporting information


**Supplementary Table S1.** Methods of population‐based studies determining the prevalence of progressive supranuclear palsy.
**Supplementary Table S2.** Methods of population‐based studies determining the prevalence of corticobasal degeneration.
**Supplementary Table S3.** Progressive supranuclear palsy overall and age restricted, sex‐ and age‐stratified crude prevalence rates per 100,000 (95% confidence interval).
**Supplementary Table S4.** Corticobasal syndrome/degeneration overall, sex‐ and age‐stratified crude prevalence rates (95% confidence interval) per 100,000.
**Appendix S1.** Search strategies.
