# Supplementary material for: Systematic Review of Prevalence Studies of Progressive Supranuclear Palsy and Corticobasal Syndrome
Source: Mov Disord Clin Pract. 2022 Jun 28;9(5):604–13. doi: 10.1002/mdc3.13489 (PMC9274340; doi:10.1002/mdc3.13489)
Supplement: Supplementary file 1 — Supplementary Table S1. Methods of population‐based studies determining the prevalence of progressive supranuclear palsy. Supplementary Table S2. Methods of population‐based studies determining the prevalence of corticobasal degeneration. Supplementary Table S3. Progressive supranuclear palsy overall and age restricted, sex‐ and age‐stratified crude prevalence rates per 100,000 (95% confidence interval). Supplementary Table S4. Corticobasal syndrome/degeneration overall, sex‐ and age‐stratified crude prevalence rates (95% confidence interval) per 100,000. Appendix S1. Search strategies. [file MDC3-9-604-s001.docx]

**Supplementary information**

**Table 1:** Methods of population based studies determining the prevalence of progressive supranuclear palsy

| **Study ID**  **Country**  **Stipulated aims** | **Population size and source** | **Prevalence day** | **Case definition**  (Inclusion/Exclusion Criteria) | **Case identification method**  Recruitment period  Response rate and refusers | **Case verification method**  Number of cases examined by a specialist  Follow up |
| --- | --- | --- | --- | --- | --- |
| Fleury, 2018  Canton of Geneva, Switzerland.  Prevalence and incidence of PD and parkinsonism. | 470,512  Office Cantonal de la Population et des Migrations. | 01/01/13 | **Inclusion:** 1996 consensus criteria^a^.  **Exclusion:** 1996 consensus criteria^b^. | Multisource: clinical records of those in University hospitals with relevant inpatient and outpatient ICD-10 diagnostic coding (multiple codes); clinical records of private neurologists, clinical records from nursing homes.  Identification period 01/01/03-31/12/12.  85% participation private neurologists, 16% of nursing homes (19.5% of population living in nursing homes). | Neurologist review of clinical notes and imaging data.  None examined.  No follow up. |
| Coyle-Gilchrist, 2016.  Cambridge-shire and Norfolk,  UK.  Prevalence and incidence of the major FTLD-associated syndromes. | 1,690,000  2013 UK Office for National Statistics Census midyear estimate. | 01/01/14 | **Inclusion:** NNIPPS Study criteria (derived from 1996 consensus criteria^a^): *s*upranuclear ophthalmoplegia and postural instability or falls (within **3** years from disease onset). For cases at a boundary between two diagnostic categories, or with an overlap of clinical features, the dominant syndrome or phenotype was used for categorisation.  **Exclusion:** Any of cerebellar ataxia, symptomatic autonomic dysfunction, or tremor at rest. | Multisource: referral of FTLD associated syndromes from regional specialist clinics (received referrals from primary, secondary, and tertiary care), specialist nurses, and self-referral; members of relevant patient charities, clinical research networks and database (to identify those no longer under review). Referral sources contacted in person, by letter, and by e-mail before and during the study.  Identification period 01/01/13 to 31/12/14. Clinical databases searched from 2003.  Refusers not described. Consent required for case notification. | Diagnostic criteria applied based on clinical interview, examination, and relevant tests including brain imaging. For cases unable or unwilling to be assessed in person, existing medical records were accessed.  Of 234 identified, 197 (84.2%) were examined by study team. Of 204 eligible cases with FTD associated syndromes, 167 (81.7%) seen by study team. Detailed information sufficient to apply diagnostic criteria in 200 (98.0%).  No follow up. |
| Takigawa, 2016.  Yonago City, Japan.  Prevalence of PSP compared to a previous report. | 148,271  Population demographics from the Yonago city office. | 01/10/10 | **Inclusion:** Richardson’s syndrome subtypes diagnosed 1996 consensus criteria^a^. Subtypes of PSP classified according to Williams et al ([2005](https://www.ncbi.nlm.nih.gov/pmc/articles/PMC5166993/#brb3557-bib-0019), [2007](https://www.ncbi.nlm.nih.gov/pmc/articles/PMC5166993/#brb3557-bib-0020))  **Exclusion:** 1996 consensus criteria^b^. | Medical records from patients at single University Hospital with diagnoses of PD, PSP, CBD, MSA, parkinsonian syndrome.  Annual survey from 2009 to 2014.  Unclear if any refusers. Not described if so. | Identified individuals underwent history, examination, and imaging (MRI, MIBG) assessment.  Percentage reviewed not explicit: “some patients with PSP were not able to be contacted for this survey”.  No follow up. |
| Withall, 2014.  Eastern Sydney, Australia.  Prevalence and causes of young onset dementia. | 129,070 30–64 years  (68,867 30–44 years,  60,203 45-64 years).  Australian Bureau of Statistics 2006 census data. | 01/06/08 | **Inclusion:** Included if alive and residing within the catchment area on the census date; clinical diagnosis of dementia; onset of memory, behavioural, and/or language symptoms before the age of 65 years; and persistent cognitive impairment for at least six months.  Diagnostic criteria used for PSP unclear. | Brief structured questionnaire to health professionals in the catchment area (multiple hospital specialities, allied health professionals, GPs) to identify patients with YOD (selection of specific aetiologies to select, including PSP). Second questionnaire sent if no response, followed by a reminder email or phone call. Memory clinics and hospital records searched for YOD patients using ICD-10 codes.  Questionnaire sent 12 months prior census date 01/06/08, hospital record ICD-10 code search 12 months prior census in two hospitals in area, memory clinics searched 5 years prior census 2003-2008  83% response rate (23% for GP’s) to initial case finding questionnaire. | Verification of diagnoses by medical case note review.  Of 239 potential cases, 141 with YOD identified. Diagnoses were veriﬁed by the medical case notes in 115 (81.6%) of 141.  Of the remaining 26 patients, 21 referred from health services and 5 from residential care facilities.  No follow up. |
| Osaki, 2011  Koban district, Japan.  Prevalence of PD and atypical parkinsonism. | 66,465  21,483 ≥65  2005 Population Census of Japan. | 01/11/07 | **Inclusion:** 1996 consensus criteria^a^.  **Exclusion:** 1996 consensus criteria^b^. | Referrals sought from clinicians in medical institutions (11 hospitals), Japanese long-term care insurance system facilities in the district (n=?), Susaki public health office. Reminder letters sent twice.  Identification period not given.  Refusers not described. | Medical records of those referred screened. Those with relevant diagnoses assessed by neurologists (standardised form, H&Y staging, medication review).  145 relevant diagnoses (PD, PSP, CBD or MSA) of 184 medical records reviewed by neurologists. Of these 145, 143 (98.6%) assessed.  No follow up. |
| Wada-Isoe, 2009.  Ama-cho, Japan.  Prevalence of dementing disorders. | 2,430  943 aged ≥65  3 public health nurses working as permanent care providers kept health information of the entire town for >20 years. | 01/03/08 | **Inclusion:** 65+, 1996 consensus criteria^a^.  **Exclusion:** 1996 consensus criteria^b^ plus cognitive decline secondary to major depression and other psychiatric disorders if proven to be the main cause for cognitive decline. | Door to door, 2 phase. Phase 1: Screening of all aged 65+ for suspected cognitive impairment by interview administered by public health nurses, compared with medical history obtained from family doctor records. Phase 2: Clinical examination by neurologists of those with cognitive impairment sufficient to impair function.  Identification period not given.  “Very high response rate” but not quantified. Refusers not described. | Clinical examination by neurologists (history, physical and cognitive examination [MMSE], functional scales) of those with cognitive impairment sufficient to impair function. Brain imaging (predominantly CT) utilised if available.  120 (100%) of cases identified in phase 1 were examined in phase 2.  No follow up. |
| Wermuth, 2008.  Faroe Islands.  Assess possible changes in prevalence and incidence of PD over time. | 48,371  Faroese Registry of Residents 2005. | 01/07/05 | **Inclusion:** 1996 consensus criteria^a^ – not explicit in text but diagnostic criteria referenced.  **Exclusion:** 1996 consensus criteria^b^.  Detailed classification of those with atypical parkinsonism not attempted when severe dementia was present. | Multisource: All Faroese residents receiving levodopa (LD) and dopamine agonists were identified from Chief Pharmacist records; cases diagnosed with PD or parkinsonism by local neurologist at the National Hospital or two regional hospitals; requests to all GPs to contact patients with PD or parkinsonism; study publicised news/media allowing self-referral.  All patients known to be receiving anti-parkinsonian medication received a letter of invitation and a follow-up telephone call. Those diagnosed at the hospitals by the neurologist and the GPs received a similar letter of invitation but no follow-up telephone call.  Pharmacy records searched 15/04/04-15/04/05. Duration of other case finding methods unclear.  For non-participating individuals, information was limited to diagnosis and the time of symptom onset from medical records. Among the 35 decliners and non-responders, one had atypical parkinsonism. | Cases identified underwent history and examination by a neurology trainee or movement disorder specialist.  Of 153 patients with possible PD or parkinsonism, 102 (67%) clinically examined. 19 declined clinical examination, 16 were non-responders, 12 excluded due to other veriﬁed neurological diagnoses and 4 died.  No follow up. |
| Barbosa, 2006  Bambuí, Brazil  Prevalence of Parkinsonism and PD. | 1,238  Complete census carried out in 1996 (source unclear) prior larger study. Eligible population in area not reassessed at time of prevalence assessment (2001). | Not stated. | **Inclusion:** 1996 consensus criteria^a^.  **Exclusion:** 1996 consensus criteria^b^**.** | Two-phase. Phase I screening questionnaire. Subjects who responded positively to at least 2 of the 9 questions or 1+ on PD medications, were selected for phase II. Phase II neurological examination by neurologist or care of the elderly physician.  Prevalence study part of larger study (Bambuí Health and Ageing Study). Prevalence parkinsonism investigated in fourth follow-up, among the survivors of the initial study population (n=1,606 of 1,742 eligible). | Examined independently by at least 2 of 4 movement disorder-trained neurologists or geriatrician. A senior movement disorder neurologist evaluated each of the suspected cases (history, examination) to make the diagnosis.  42 of 709 (5.9%) screening positive in phase I were not examined in phase II due to participation refusal or death.  No mention of follow-up in relation to determination of prevalence. |
| Kawashima, 2004.  Yonago  City, Japan  Prevalence of PSP. | 137,420  Source of population size not explicitly stated. | 01/04/99 | **Inclusion:** 1996 consensus criteria^a^.  **Exclusion:** 1996 consensus criteria^b^. | Review of all medical records in the university hospital (containing neurology department) for relevant diagnoses, referrals requested from visiting neurologists attending remaining 4 general hospitals in area, screening (examination for parkinsonism with dementia, gaze palsy) in 10 registered nursing homes.  Surveyed twice over a 3-year period, in 1999 and in 2002.  Total nursing home population unclear, reasons if/for refusal not stated. | Cases from medical records/neurologist referrals were not re-examined. Sampled nursing home residents examined.  956 patients in nursing home in 1999 and 732 patients in 2002 were examined.  No follow up. |
| Zhang, 2003.  Greater Beijing, China- 12 urban and 15 rural areas randomly selected.  Prevalence of PD to compare to previous studies in Chinese populations. | 5,974 (5952 at home, 22 in institutions)  Registry ofﬁce of each community. | 31/12/97 | **Inclusion:** Persons ≥55, registered in the community and present at the time of data collection (or within a month before that time).  Persons registered in the community but living elsewhere in a chronic-care institution also deemed eligible. Persons not registered in the community were eligible if, at the time of data collection, they were living in the community for at least 1 month with a ﬁrst-degree relative registered there.  Parkinsonism characterized by two or more of: resting tremor, bradykinesia, rigidity, and impaired postural reﬂexes.  PSP Collins neuropath based clinical criteria referenced.  **Exclusion:** <55 years. | Community leaders promoted study at civic meetings and posted ﬂyers in neighbourhoods where data collection was taking place.  Questionnaire and examination offered to all eligible participants (unclear if all over 55). CT/MRI also available in a proportion (52% of those with clinical non-PD parkinsonism). Up to 3 attempts made to locate and contact each eligible person residing in the study area. Attempts also made to trace eligible persons who lived in chronic-care institutions away from the study area.  Preliminary examinations and diagnoses October 1996 to October 1997.  Of 231 (4%) not examined- 46 refused, 9 unreachable in a nursing home, 21 unreachable in a hospital, 155 not at home. | 96% of total eligible population examined at least once. 144 with parkinsonism identified. 78% underwent one or more additional neurological examinations.  Preliminary diagnosis of PD, non-PD parkinsonism, or suspected parkinsonism (one cardinal sign and the equivocal presence of another) were followed for several months or more before a ﬁnal diagnosis was made. Persons with suspected parkinsonism or questionable diagnoses were followed more closely, with repeated visits to their homes or with a priority admission to the Peking Union Medical College (PUMC) Hospital in Beijing for further evaluation. Overall follow-up 12 to 53 months (median 40 months). |
| Yamada, 2001.  Ama-cho, Japan.  Prevalence of dementing disorders. | 16,798  3,715 ≥65  3 public health nurses working as permanent care providers kept health information of the entire town for >20 years. | 01/01/98 | **Inclusion:** ≥65. Diagnostic criteria PSP not stated.  **Exclusion:** Cognitive decline secondary to major depression and other psychiatric disorders if proven to be the main cause for cognitive decline. | Phase 1: Screening for cognitive impairment in individuals ≥65, interview administered by public health nurses, compared with medical history. Phase 2: Clinical examination by neurologists. MRI also utilised where available.  Phase 1: one month duration (January 1998)  Refusers not described. | Clinical examination by neurologists (physical and cognitive examination-MMSE). MRI utilised where available.  Numbers examined not stated. Not all as paper states that in those who could not examined directly, information was collected from family, nurses and doctors in the town.  No follow up. |
| Nath, 2001.  UK, North- East England, and Newcastle.  Point prevalence of PSP. | National: 59,236,500  Regional: 2,589,240  Community: 259,998  Mid-1998 census figures for UK population for national and regional study. Newcastle and North Tyneside Health Authority for general practices population in community study. | 01/01/99 | **Inclusion:** 1996 consensus criteria^a^.  **Exclusion:** In community study excluded if used parkinsonian medication for other indications, if miscoded, or if symptom onset within 6 months of use of dopamine blocking agent. 1996 consensus criteria^b^. | National study: Passive multisource: surveillance unit, patient charities, Office of National Statistics, consultant neurologist referral (those with interest in movement disorders particularly targeted).  Regional: Active multisource: clinician referral (neurologist, care of the elderly, old age psychiatry), unselected outpatient correspondence review in same key specialities, variety of clinical database screens, search of ICD-10 diagnostic coding of inpatients.  Community: 2 phase. Phase 1: Keyword search of diagnostic and therapeutic registers from a representative sample of 42 approached GP practices. Phase 2: Interview and examination if parkinsonism and either absent/unsustained response to LD or additional atypical clinical features. Reminder letters sent to patients who did not reply. If they failed to respond they were telephoned.  Not clear over what time period all referrals requested/ cases sought. Regional correspondence reviewed from December 1998 to January 2000.  83% GP practice participation. Consenting and non consenting practices had similar age and sex distributions. Patient refusers not described at national/regional level. In community study those not examined were significantly older (p<0.005). | National: Medical notes review only. 34% medical records available for review.  Regional: Diagnosis based on review of medical records, but examined where possible. 75% examined. Medical notes of 3% referred could not be reviewed.  Community: In phase 1 records available for 397 (81%) of 490 identified as eligible. In phase 2, 82.3% examined.  No follow-up of diagnosed cases. In regional study where correspondence indicated parkinsonism, atypical features, unexplained falls, or unexplained eye movement disorder, a further 6 month correspondence was invited from clinicians to identify emerging diagnoses. |
| Schrag, 1999.  London and Kent, UK.  Population prevalence of PSP and MSA. | 121,608  14,272 ≥65  12 general practices participating in a linkage scheme with National Hospital plus three additional practices chosen – to be eligible had to have computerised records, be located in area typical of overall population, and not be involved in other population based studies.  Numbers of patients registered with each GP practice, stratified by sex and age, obtained from the practice or the relevant Family Health Services Authority. | 01/07/97 | **Inclusion:** 1996 consensus criteria^a^.  **Exclusion:** 1996 consensus criteria^b^  At screening, excluded patients who had only tremor with onset at younger than 50 years.In review of records identified by screening excluded if: used parkinsonian medication for other indications, had a known alternative cause of tremor (e.g. hyperthyroidism)’ onset of parkinsonian symptoms within 6 months of at least 6 months' treatment with dopamine receptor blocking drugs, developed dementia before the onset of parkinsonism, or records miscoded. | In 12 sampled GP practices, computerised records screened for patients with the entries: “Parkinson's disease”, “parkinsonism”, “progressive supranuclear palsy”, “striatonigral degeneration”, “Shy-Drager syndrome”, “parkinsonism with orthostatic hypotension”, and “other extrapyramidal disorders”, as well as “tremor” if onset was at older than 50 years. Also included patients who had ever been prescribed antiparkinsonian drugs.  Identified patients invited to be seen (interview, examination, video). Reminder letters sent to patients who did not reply and, if they failed to respond, they were telephoned.  Identification period unclear.  Patients not examined were significantly older than those examined (79.5 years vs 73.0 years, p<0.001). No sex differences. | 679 selected by screening, 241 eligible after review of records. Of these, 202 (84%) assessed.  For patients who refused to participate information from GP’s and hospitals sought.  All patients with a diagnosis of parkinsonism, PSP, or MSA were asked to complete questionnaires every 3 months on the development of atypical features and symptoms of progression. At the end of the study GP’s were asked whether they had noticed any new atypical features in any of their patients. Patients with no definitive diagnosis or with atypical features at the first visit, or who developed them during follow-up, after at least 1 year were also reassessed. |
| Wermuth,1997.  Faroe Islands.  Determine whether high levodopa use indicates a high prevalence of PD. | 43,709  Faroe Statistic Bureau. | 01/07/95 | **Inclusion:** Not specified for PSP.  **Exclusion:** Excluded those who had died before the prevalence date, were incorrectly registered, were treated with LD for other diseases, or had “other diseases” (?including other causes parkinsonism).  Those with severe dementia at examination excluded. | Multi-source: cases taking LD or dopamine agonist medication from Faroe Islands pharmacies; registered patients with suspected PD in the National Hospital (only hospital with a neurologist); information on patients with possible parkinsonism from all GPs; all nursing homes visited by neurologist who interviewed the staff on possible patients with parkinsonism.  Information medication 01/11/93 to 15/06/95. Patients registered with neurologist 01/01/90 to 31/12/94.  Only patients thought to have PD interviewed and examined. | 122/124 (98.4%) examined. Remaining 2 cases had been previously examined neurologist.  No follow up. |
| de Rijk,1995.  Ommoord, Rotterdam, Netherlands.  Prevalence of PD. | 10,275 ≥55  Names and addresses drawn from the municipal register. | Not stated. | **Inclusion:** “Definite” parkinsonism if at least two of the cardinal signs present in a subject not taking antiparkinsonian drugs, or in a subject treated with antiparkinsonian medication, one or more signs had improved by treatment.  “Possible” parkinsonism if only one sign present in an untreated subject or if a specifically treated patient did not report benefit from treatment and had only one cardinal sign. Possible parkinsonism not included in the prevalence figures.  Irrespective of the results of screening,  reviewed the medical records of demented institutionalized participants to check for a previous diagnosis of PD or secondary parkinsonism.  Inclusion/Exclusion not specified for PSP. | All inhabitants of Ommoord, ≥55 years, living independently or institutionalized, were invited to participate by mail and were contacted by telephone 2 weeks later.  Two-phase design to assess prevalence PD. In the first phase, all participants asked about previous diagnosis of PD, and any drug use coded according to the Anatomical Therapeutic Chemical classification index. Every participant neurologically examined by one of the study physicians. All subjects who used antiparkinsonian drugs, reported that they had PD, or had at least one possible cardinal sign of parkinsonism (i.e. resting tremor, rigidity, bradykinesia, or impaired postural reflexes) at the neurologic screening examination were invited for further evaluation in a second phase. In the second phase, those screening positive were assessed by a neurologist or neurology trainee (history and examination).  Cross sectional survey started in 1990 and completed June 1993. | 7,983 (78%) agreed to participate Rotterdam study and signed informed consent statements. The number who visited the research centre decreased slightly, to 7,129 (69%), due to refusal, disease, or death. For the prevalence study 6,969 subjects (**68%**) who had a neurologic screening examination, who were using antiparkinsonian medication or reported that they had PD included.  653 (9.4%) of 6,969 screened positive and invited for second phase evaluation. In second phase 499 (76%) of 653 clinically examined. In 128 of the 154 subjects who screened positive and who could not be further examined, obtained a sufficient amount of other medical information on which to base the diagnosis.  All newly diagnosed patients with definite parkinsonism were re-evaluated by a second neurologist.  No follow up. |
| Golbe, 1988.  Middlesex and Somerset Counties,  New Jersey, USA.  PSP prevalence and sex ratio. | 799,022  County population data from the 1980 US Census | 01/05/86 | **Inclusion:** All of the following: Onset after age 40, progressive course, bradykinesia, supranuclear gaze palsy. Plus any three of: dysarthria or dysphagia, axial rigidity greater than limb rigidity, neck in a posture of extension, tremor minimal or absent, frequent falls or gait disturbance early in disease course, pyramidal tract signs.  **Exclusion:** Early or prominent cerebellar signs, unexplained polyneuropathy, dysautonomia other than isolated postural hypotension which was not iatrogenic. | Neurologist referral (50 clinicians) of cases seen in last 5 years (non-responders contacted by phone), announcements at meetings of local PD support groups, in their newsletters, sought nursing director of every nursing home listed in the telephone directories of relevant counties.  Referrals sought for cases seen 5 years prior study onset. | Examination, standardised history with relative input, video.  48 (96%) of 50 cases identified were examined to verify the diagnosis. Remaining 2 cases previously examined and diagnosed by neurologists.  No follow up. |

CBD corticobasal degeneration; CT computerised tomography; GP general practitioner; H&Y Hoehn and Yahr scale; ICD-10 International Classification of Disease Version 10; LD levodopa; MMSE Mini Mental State Examination; PD Parkinson’s disease; PSP progressive supranuclear palsy; FTD frontotemporal dementia; MIBG meta-iodobenzylguanidine; MRI magnetic resonance imaging; MSA multiple system atrophy; FTLD frontotemporal lobar degeneration; YOD young onset dementia; ^a^Litvan Inclusion Criteria. Possible PSP (1) Gradually progressive disorder, (2) Onset at age 40 or later, (3) Either vertical (upward or downward gaze) supranuclear palsy or both slowing of vertical saccades and prominent postural instability with falls in the first year of disease onset, (4) No evidence of other diseases that could explain the foregoing features, as indicated by mandatory exclusion criteria. Probable PSP (1) Gradually progressive disorder, (2) Onset at age 40 or later, (3) Vertical (upward or downward gaze) supranuclear palsy and prominent postural instability with falls in the first year of disease onset, (4) No evidence of other diseases that could explain the foregoing features, as indicated by mandatory exclusion criteria. ^b^Litvan Exclusion Criteria: Recent history of encephalitis; alien limb syndrome, cortical sensory deficits, focal frontal or temporoparietal atrophy; hallucinations or delusions unrelated to dopaminergic therapy; cortical dementia of Alzheimer’s type; prominent early cerebellar symptoms or prominent early unexplained dysautonomia; severe asymmetric parkinsonian signs; neuroradiological evidence of relevant structural abnormality, Whipple’s disease.

**Table 2:** Methods of population based studies determining the prevalence of corticobasal syndrome

| **Study ID**  **Country**  **Stipulated aims** | **Population size and source** | **Prevalence day** | **Case definition**  (Inclusion/Exclusion Criteria) | **Case identification method**  Recruitment period  Response rate and refusers | **Case verification method**  Number of cases examined by a specialist  Follow up |
| --- | --- | --- | --- | --- | --- |
| Fleury,  2018 | 470,512  Office Cantonal de la Population et des Migrations | 01/01/13 | **Inclusion:** Armstrong criteria^a^.  **Exclusion:** Not explicit if biomarker exclusion criteria in Armstrong criteria applied. | Multisource: clinical records of those in University hospitals with relevant inpatient and outpatient ICD-10 diagnostic coding (multiple codes); clinical records of private neurologists, clinical records from nursing homes.  85% participation private neurologists, 16% of nursing homes (19.5% of population living in nursing homes).  01/01/03-31/12/12. | Neurologist review of clinical notes and imaging data.  None examined.  No follow up. |
| Coyle-Gilchrist, 2016.  Cambridge-shire and Norfolk,  UK.  Prevalence and incidence of the major FTLD-associated syndromes. | 1,690,000  2013 UK Office for National Statistics Census midyear estimate. | 01/01/14 | **Inclusion:** Armstrong criteria^a^.  For cases at a boundary between two diagnostic categories, or with an overlap of clinical features, the dominant syndrome or phenotype was used for categorisation.  **Exclusion:** Any of cerebellar ataxia, symptomatic autonomic dysfunction, or tremor at rest. Not explicit if biomarker exclusion criteria in Armstrong criteria applied. | Multisource: referral of FTLD associated syndromes from regional specialist clinics (received referrals from primary, secondary, and tertiary care), specialist nurses, and self-referral; members of relevant patient charities, clinical research networks and database (to identify those no longer under review). Referral sources contacted in person, by letter, and by e-mail before and during the study.  Identification period 01/01/13 to 31/12/14. Clinical databases searched from 2003.  Refusers not described. Consent required for case notification. | Diagnostic criteria applied based on clinical interview, examination, and relevant tests including brain imaging. For cases unable or unwilling to be assessed in person, existing medical records were accessed.  Of 234 identified, 197 (84.2%) were examined by study team. Of 204 eligible cases with FTD associated syndromes, 167 (81.7%) seen by study team. Detailed information sufficient to apply diagnostic criteria in 200 (98.0%).  No follow up. |
| Khedr, 2015.  Qena, Upper Egypt.  Explore the high prevalence rate of PD reported in previous Egyptian studies. | 8,760  Simple random sample of 10 areas from Qena Governorate.  Subsequent systematic random sample of households in areas sampled (every third household, family next door if refusal)  Central Agency for Public Mobilization and Statistics, Government of Egypt 2011 and 2012 referenced.for total Qena population. | 31/08/13 | **Inclusion:** diagnosis made on the basis of DSM- IV criteria (core features of progressive asymmetrical akinetic-rigid syndrome, higher cortical signs, ideomotor apraxia, postural/action tremor, unilateral dystonia, unresponsiveness to LD listed). | Multi-stage sampling: Phase 1 PD screening questionnaire applied in community. Phase 2: clinical evaluation.  Survey period 01/09/11 to 31/08/13  733 of sample population were away at the time of survey. 40 families refused to participate- each replaced by the family next door. | Clinical evaluation by a neurologist including history, examination, bloods, neuroimaging (CT, MRI) and EEG.  Fifty-one subjects who screened positive were selected and referred to hospital to be fully evaluated and investigated.  No follow up. |
| Withall, 2014.  Eastern Sydney, Australia.  Prevalence and causes of young onset dementia. | 129,070 30–64 years  (68,867 30–44 years,  60,203 45-64 years).  Australian Bureau of Statistics 2006 census data. | 01/06/08 | **Inclusion:** Included if alive and residing within the catchment area on the census date; clinical diagnosis of dementia; onset of memory, behavioural, and/or language symptoms before the age of 65 years; and persistent cognitive impairment for at least six months.  Diagnostic criteria used for CBD unclear. | Brief structured questionnaire to health professionals in the catchment area (multiple hospital specialities, allied health professionals, GPs) to identify patients with YOD (selection of specific aetiologies to select, including PSP). Second questionnaire sent if no response, followed by a reminder email or phone call. Memory clinics and hospital records searched for YOD patients using ICD-10 codes.  Questionnaire cases seen 12 months prior census date 01/06/08, hospital record ICD-10 code search 12 months prior census in two hospitals in area, memory clinics searched 5 years prior census 2003-2008  83% response rate (23% for GP’s) to initial case finding questionnaire. | Verification of diagnoses by medical case note review.  Of 239 potential cases, 141 with YOD identified. Diagnoses were veriﬁed by the medical case notes in 115 (81.6%) of 141.  Of the remaining 26 patients, 21 referred from health services and 5 from residential care facilities.  No follow up. |
| Osaki, 2011.  Koban district, Japan.  Prevalence of PD and APS. | 66,465  21,483 ≥65  2005 Population Census of Japan. | 01/11/07 | **Inclusion:** Kumar criteria^b^ | Referrals sought from clinicians in medical institutions (11 hospitals), Japanese long-term care insurance system facilities in the district (n=?), Susaki public health office. Reminder letters sent twice.  Identification period not given.  Refusers not described. | Medical records of those referred screened. Those with relevant diagnoses assessed by neurologists (standardised form, H&Y staging, medication review).  145 relevant diagnoses (PD, PSP, CBD or MSA) of 184 medical records reviewed by neurologists. Of these 145, 143 (98.6%) assessed.  No follow up. |
| Wermuth, 2008.  Faroe Islands.  Assess possible changes in prevalence and incidence of PD over time. | 48,371  Faroese Registry of Residents 2005. | 01/07/05 | **Inclusion:** Lang criteria^c^.  **Exclusion:** Detailed classification of those with atypical parkinsonism not attempted when severe dementia was present. Lang criteria^c^. | Multisource: All Faroese residents receiving levodopa (LD) and dopamine agonists were identified from Chief Pharmacist records; cases diagnosed with PD or parkinsonism by local neurologist at the National Hospital or two regional hospitals; requests to all GPs to contact patients with PD or parkinsonism; study publicised news/media allowing self-referral.  All patients known to be receiving anti-parkinsonian medication received a letter of invitation and a follow-up telephone call. Those diagnosed at the hospitals by the neurologist and the GPs received a similar letter of invitation but no follow-up telephone call.  Pharmacy records searched 15/04/04-15/04/05. Duration of other case finding methods unclear.  For non-participating individuals, information was limited to diagnosis and the time of symptom onset from medical records. Among the 35 decliners and non-responders, one had atypical parkinsonism. | Cases identified underwent history and examination by a neurology trainee or movement disorder specialist.  Of 153 patients with possible PD or parkinsonism, 102 (67%) clinically examined. 19 declined clinical examination, 16 were non-responders, 12 excluded due to other veriﬁed neurological diagnoses and 4 died.  No follow up. |
| Barbosa, 2006  Bambuí, Brazil  Prevalence of Parkinsonism and PD. | 1,238  Complete census carried out in 1996 (source unclear) prior larger study. Eligible population in area not reassessed at time of prevalence assessment (2001). | Not stated. | **Inclusion:** Unclear, 1996 PSP consensus criteria stated but different paper referenced. | Two-phase. Phase I screening questionnaire. Subjects who responded positively to at least 2 of the 9 questions or 1+ on PD medications, were selected for phase II. Phase II neurological examination by neurologist or care of the elderly physician.  Prevalence study part of larger study (Bambuí Health and Ageing Study). Prevalence parkinsonism investigated in fourth follow-up, among the survivors of the initial study population (n=1,606 of 1,742 eligible). | Examined independently by at least 2 of 4 movement disorder-trained neurologists or geriatrician. A senior movement disorder neurologist evaluated each of the suspected cases (history, examination) to make the diagnosis.  42 of 709 (5.9%) screening positive in phase I were not examined in phase II due to participation refusal or death.  No mention of FU in relation to determination of prevalence. |
| Tan, 2004.  Ang Mo Kio, Bishan,Toa Payoh, Serangoon, and Yishun districts of Singapore.  Prevalence of PD among Singapore Chinese, Malay, and Indian middle-aged and elderly adults. | Resident population 689,756 of whom 152,869 aged 50 and above. Targeted disproportionate random sample of 25,000 individuals (60% Chinese, 20% Malay, 20% Indian).  Sample 24,978 of which 22,279 eligible at ≥50.  Ministry of Home Affairs database used to identify those over 50. | 02/04/2001 | **Inclusion**: Kumar criteria^b^ | Three-phase design. Phase 1: door-to-door survey using a validated 10-question questionnaire for parkinsonism. Phase 2: examination of those who screened positive. Phase 3: those with suspected PD or on medication to treat PD examined by a movement disorders specialist.  Phase 1: 7373 of 22279 eligible refused or uncontactable (67% participation).  Identification period not given.  The mean age of participants (63.43 years) was higher than non-participants (62.87 years, p=0.0001). The participation rate Chinese 64%, 70% Malays, and 74% Indians (p < 0.0001). | Clinical examination or medical record review.  Phase 2: 1,074 of 1,258 (85.4%) screening positive examined (neurology, care of elderly or other medical doctors). Of those not examined, medical records reviewed in 172, 12 not evaluated (1 death, 4 refusal, 5 moved, 2 uncontactable). Phase 3: 80 of 94 (85.1%) examined phase 3 (8 died, 1 refused, 2 moved, 3 uncontactable).  No follow up. |
| Harvey, 2003.  Kensington, Chelsea, Westminster and Hillingdon, London, UK  Prevalence of dementia. | 567,500  2001 UK Census | “Census day” not stated. | Inclusion: Diagnostic criteria for CBD not explicit, “other dementias for which DSM-IV criteria are available”. Mixed diagnoses avoided with single diagnosis reached by consensus of two authors. | Multisource: Clinician (GP, neurology, psychiatry, care of the elderly, general medicine) and practitioner (social services, day centres, home care, community care teams) referral of any case with dementia onset <65 years under their care on census day; hospital information systems and case registers searched for ICD 9/10 diagnoses; hand searching of copies of clinic letters, discharge summaries, and departmental databases in neurology and psychiatry.  Identification period not given.  Refusers not described. | Case note review or examination.  Half of those identified by case note review approached to be examined.  Of 185 cases confirmed by medical record review, 87 (47%) underwent clinical assessment (consent required).  No follow up. |
| Trenkwalder, 1995? |  |  |  |  |  |

^a^Armstrong criteria: (1) Presentation: insidious onset and gradual progression, (2) Minimum duration of symptoms: 1 year, (3) Age at onset ≥50 years, (4) Permitted phenotypes: (a) probable corticobasal syndrome or (b) fronto-behaviour-spatial syndrome or nonfluent/agrammatic variant of primary progressive aphasia plus at least one corticobasal feature. A family history of 2 or more affect relatives or a genetic mutation affecting tau protein (e.g. MAPT) are exclusion criteria for probable sporadic CBD. The clinical research criteria for possible CBD are: (1) Presentation: insidious onset and gradual progression, (2) Minimum duration of symptoms: 1 year, (3) Permitted phenotypes: (a) possible corticobasal syndrome or (b) fronto-behaviour-spatial syndrome or nonfluent/agrammatic variant of primary progressive aphasia or (c) progressive supranuclear palsy syndrome plus at least one corticobasal feature. There is no minimum age for possible CBD and a family history and genetic mutation are permitted; ^b^Kumar criteria. Inclusion: Core features: chronic progressive course, asymmetric at onset (includes speech dyspraxia, dysphasia). Presence of: higher cortical dysfunction (apraxia, cortical sensory loss, alien limb) and movement disorder (akinetic-rigid syndrome resistant to levodopa, and limb dystonia or spontaneous and reflex focal myoclonus). Exclusion: early dementia, early vertical gaze palsy, rest tremor, severe autonomic disturbances, sustained responsiveness to levodopa, lesions on imaging studies indicating another pathologic process is responsible; ^c^Lang criteria. Inclusion: rigidity plus one cortical sign (apraxia, cortical sensory loss or alien limb phenomenon) or asymmetric rigidity, dystonia and focal reflex myoclonus. Exclusion criteria: early dementia, vertical gaze palsy, rest tremor, severe autonomic disturbances, sustained responsiveness to levodopa, lesions on imaging studies indicating another pathologic process is responsible.

**Table 3**: Progressive supranuclear palsy overall and age restricted, sex- and age- stratified crude prevalence rates per 100,000 (95% CI)

| **Study ID and population size** |  | **Case frequency** | | | **Crude prevalence (95% CI) per 100,000** | | | | **Age stratified crude prevalence per 100,000 (95% CI)**  **[number of cases]** | | | | | | | **Age at onset (years)**  **(Mean (SD))** |
| --- | --- | --- | --- | --- | --- | --- | --- | --- | --- | --- | --- | --- | --- | --- | --- | --- |
|  |  | **Total** | **Male** | **Female** | **Overall** | **Male** | **Female** | | **40–49**  **years** | | **50–59**  **years** | **60–69**  **years** | **70–79**  **years** | **80-89**  **years** | **90+**  **years** |  |
| **Fleury, 2018**  470,512  (227,697M/242,815F) | Overall | 39 | 22 | 17 | 8.3 (5.9,11.3) | 9.7 (6.1,14.6) | 7.0 (4.1,11.2) | | 0.0  (0.0,5.0) | | 5.0  (1.0,14.5)  [3] | 15.1  (6.1, 31.1)  [7] | 50.2  (28.7, 81.5) [16] | 72.3  (38.5,123.6)  [13] | 0.0  (0.0, 90.3)  [0] | - |
| **Coyle-Gilchrist, 2016**  1,690,000 | Overall | 48 | 29 | 19 | 2.8 (2.1, 3.8) | 1.7 | 1.1 | | 0.0 | | 1.8  [50-54 years]  3.0  [55-59 years] | 6.0  [60-64 years]  11.4  [65-69 years] | 18.7  [70-74 years]  11.3  [75-79 years] | 6.4  [80-84  years]  2.2  [85-89  years] | 2.9 | Assessment 72.6 (7.8)  Onset to assessment 4.7 (3.5) |
| **Takigawa, 2016**  139,683 (66,482M/73,200F) | Overall    RS total  Probable-RS Possible-RS  PSP‐P  PSP‐PAGF | 25  20  16  4  3  2 | 12  7  4  3  3  2 | 13  13  12  1  0  0 | 17.9 (11.6, 26.4)^†^  14.3 (8.7, 22.1)^†^  11.5 (6.5, 18.6)  2.9 (0.8, 7.3)  2.1 (0.4, 6.3)  1.4 (0.2, 5.2) | 18.0 (9.3, 31.5)^†^  10.5 (4.2, 21.7)^†^  6.0 (1.6, 15.4)  4.5 (0.9, 13.2)  4.5 (0.9, 13.2)  3.0 (0.4,10.9) | 17.8 (10.4, 30.4)^†^  17.8 (10.4, 30.4)^†^  16.4 (8.5, 28.6)  1.4 (0.0, 7.6)  0.0 (0.0, 5.0)  0.0 (0.0, 5.0) | | **-** | | **-** | **-** | **-** | **-** | **-** | 72.5 (6.9)  Assessment 77.5 (6.5)  73.6 (7.0)  74.7 (6.4)  69.0 (8.2)  76.0 (7.1)  76.0 (1.4) |
| **Osaki, 2011**  66,465 (31,805M/34,660F)  21,483 ≥65 (8,745M/12,738F) | RS total  Probable-RS  Possible-RS  ≥65 | 12  4  8  12 | 8  -  -  8 | 4  -  -  4 | 18.1 (9.3, 31.5)^†^  6.0 (1.6, 15.4)  12.0 (5.2, 23.7)  55.9 (28.8, 97.6)^†^ | 25.2 (10.9,49.6)^†^  -  -  91.5 (39.5,180.3)^†^ | 11.5 (3.1, 29.5)^†^  -  -  31.4 (8.6, 80.4)^†^ | | 0  - | | 0  - | 0  - | 4  [5]  - | 5  [6]  - | 1  [1]  - | 76 (7)  Assessment 81 (5) |
| **Wermuth, 2008**  48,371 | Overall | 2 | - | - | 4.1 (0.5, 14.9) | - | - | | **-** | | **-** | **-** | **-** | **-** | **-** | **-** |
| **Kawashima, 2004**  137,420 | RS total  Probable-RS  Possible-RS | 8  2  6 | 6  -  - | 2  -  - | 5.8 (2.5, 11.5)^†^  1.5 (0.2, 5.3)  4.4 (1.6, 9.5) | 9.1 (1.8, 16.5)  -  - | 2.8 (-1.1, 6.7)  -  - | | **-** | | **-** | **-** | **-** | **-** | **-** | 71.4 (6.6)  Assessment 74 (6) |
| **Nath, 2001**  National 59,236,500  (29,128,391M/  30,108,109F)  Regional 2,589,240  (1,268,187M/  1,321,053F)  Community 259,998  (129,383M/130,615F) | National total  Clinically verified  Probable-RS  Possible-RS  Regional total  Probable-RS  Possible-RS  Community total  Probable-RS  Possible-RS | 577  187  108  79  80  50  30  17  11  6 | 296  91  -  -  31  -  -  8  -  - | 285  96  -  -  49  -  -  9  -  - | 1.0 (0.9, 1.1)  0.3 (0.3, 0.4)  0.2 (0.1, 0.2)  0.1 (0.1, 0.2)  3.1 (2.4, 3.8)  1.9 (1.4, 2.5)  1.2 (0.8, 1.7)  6.5 (3.8, 10.5)^†^  4.2 (2.1, 7.6)  2.3 (0.8, 5.0) | 1.0 (0.9, 1.1)  (2.5, 3.8)  -  -  2.4 (1.6, 3.3)  -  -  6.2 (2.7, 12.2)^†^  -  - | 1.0 (0.8, 1.1)  (2.6, 3.8)  -  -  3.7 (2.7, 4.8)  -  -  6.9 (3.2,13.1)^†^  -  - | | **-** | | **-** | **-** | **-** | **-** | **-** | 66 (41, 83) in records reviewed*  69 (48, 83)*  67 (48, 83)* |
| **Schrag, 1999**  121,608 | RS total  Probable-RS  Possible-RS | 6  5  1 | 3  -  - | 3  -  - | 4·9 (1.8, 10.7)  4.1 (1.3, 9.6)  0.8 (0.0, 4.6) | 5·0  -  - | 4·8  -  - | | **-** | | [1] | [1] | [3] | [1] | **-** | 68.5  Assessment 72.8 |
| **Wermuth, 1997**  43,709 | Overall | 2 | - | - | 4.6 (0.6, 16.5) | - | - | | **-** | | **-** | **-** | **-** | **-** | **-** | **-** |
| **Golbe,1988**  799,022 | Overall | 11 | 6 | 5 | 1.4 (0.7, 2.5) | - | - | | **-** | | **-** | **-** | **-** | **-** | **-** | 62.9 (6.4)^‡^ |
| **Age restricted** | | | | | | | | | | | | | | | | |
| **Withall, 2014** (30–64 years)  129,070 | Overall | 3 | 2 | 1 | 2.3 (0.5, 6.8) | - | | - | | **-** | **-** | **-** | **-** | **-** | **-** | **-** |
| **Wada-Isoe, 2009** (≥65 years)  943 (386M/557F) | Overall | 2 | 2 | 0 | 212.1 (25.7, 766.1) | 518.1  (62.7, 1871.7) | | 0.0 (0.0, 662.3) | | **-** | **-** | 0 | 0 | 727.3 [2] | 0 | - |
| **Barbosa, 2006** (>60 years)  1,238 | Overall | 0 | - | - | 0.0 (0.0, 298.0) | - | | - | | - | - | - | - | - | - | - |
| **Zhang, 2003** (≥55 years)  5,974 | Overall | 1 | 0 | 1 | 16.7 (0.4, 93.3) | - | | - | | **-** | **-** | **-** | **-** | **-** | **-** | **-** |
| **Yamada, 2001**  (≥65 years)  3,715 (1,503M/2,213F) | Overall | 1 | - | - | 26.9 (0.7, 150.0) | - | | - | | **-** | **-** | **-** | **-** | **-** | **-** | **-** |
| **de Rijk,1995** (≥55 years)  10,275 (≥55) | Overall | 1 | - | - | 9.7 (0.2, 54.2) | - | | - | | - | - | - | - | - | - | - |

M male; F male; RS Richardson’s syndrome; ^†^ Numbers differ slightly from numbers stated in paper *[Median (range)],^‡^unclear if mean is 50 patients identified or 11 prevalent cases.

**Table 4:** Corticobasal syndrome/degeneration overall, sex- and age- stratified crude prevalence rates (95% CI) per 100,000

| **Study ID and population size** |  | **Case frequency** | | | **Crude prevalence (95% CI) per 100,000** | | | **Age stratified crude prevalence per 100,000 (95% CI)**  **[case frequency]** | | | | | | **Age at onset (Mean (SD))** |
| --- | --- | --- | --- | --- | --- | --- | --- | --- | --- | --- | --- | --- | --- | --- |
|  |  | **Total** | **Male** | **Female** | **Overall** | **Male** | **Female** | **40–49**  **years** | **50–59**  **years** | **60–69**  **years** | **70–79**  **years** | **80-89**  **years** | **90+**  **years** |  |
| **Fleury, 2018**  470,512 | Overall | 14 | 6 | 8 | 3.0 (1.6, 5.0) | 2.6 (1.0, 5.7) | 3.3 (1.4, 6.5) | 0.0  (0.0,5.0) [0] | 1.7  (0.0,9.2) [1] | 8.6 (2.4,22.1)  [4] | 15.7  (5.1, 36.6)  [5] | 22.2  (6.1, 57.0)  [4] | 0.0  (0.0, 90.3)  [0] | - |
| **Coyle-Gilchrist, 2016**  1,690,000 | Overall | 38 | - | - | 2.2 (1.6, 3.1) | - | - | 0.0 | 1.8  [50-54 years]  6.0  [55-59 years] | 6.0  [60-64 years]  9.5  [65-69 years] | 5.3  [70-74 years]  11.3  [75-79 years] | 8.5  [80-84  years]  0.0  [85-89  years] | 2.4 | Assessment 70.8 (8.5)^‡^  Onset to assessment 4.4 (2.7)^‡^ |
| **Khedr, 2015**  8027 | Overall | 2 | - | - | 24.9 (3.0, 90.0)^†^ | - | - | - | - | - | - | - | - | - |
| **Osaki, 2011**  66,465 (31,805M/34,660F)  21,483 ≥65 (8,745M/12,738F) | Overall  ≥65 | 6  5 | 5  4 | 1  1 | 9.0 (3.3, 19.6)^†^  23.3 (7.6, 54.3) | 15.7 (5.1, 36.7)  45.7 (12.5,117.1) | 2.9 (0.1, 16.1)  7.9 (0.2, 43.7) | 0  - | 0  - | 2  [2]  - | 2  [2]  - | 2  [2]  - | 0  - | 71 (9)  Assessment =75(9) |
| **Wermuth, 2008**  48,371 | Overall | 0 | - | - | 0.0 (0.0, 7.6) | - | - | - | - | - | - | - | - | - |
| **Age restricted** |  |  |  |  |  |  |  |  |  |  |  |  |  |  |
| **Withall, 2014** (30–64 years)  129,070 | Overall | 1 | 1 | 0 | 0.8 (0.0, 4.3) | - | - | **-** | **-** | **-** | **-** | **-** | **-** |  |
| **Barbosa, 2006** (>60 years)  1,742 | Overall | 0 | - | - | 0.0 (0.0, 211.8) | - | - | - | - | - | - | - | - |  |
| **Tan, 2004** (≥50 years)  22,279 | Overall | 1 | - | - | 4.5 (0.1, 25.0) | - | - | **-** | **-** | **-** | **-** | **-** | **-** |  |
| **Harvey, 2003** (30-64 years)  240,766 (119,839M/120,927F) | Overall | 2 | - | - | 0.8 (0.1, 3.0) | - | - | **-** | **-** | **-** | **-** | **-** | **-** |  |

‡All 48 identified cases

**Appendix 1 Search Strategies**

**1. Primary studies of prevalence of PSP or CBS**

The electronic search strategies for primary prevalence studies are as follows, including the numbers of results for each search string.

- 1. **Ovid Medline**

**03/07/17**

1. exp Supranuclear Palsy, Progressive/ 2129
2. progressive supranuclear pals$.tw,mp. 2982
3. Richardson$ syndrome.tw,mp. 65
4. Richardson's Syndrome/ 2129
5. Richardsons syndrome/ 0
6. steele richardson olszewski disease.tw,mp. 16
7. steele richardson olszewski disease/ 2129
8. steele richardson olszewski syndrome.tw,mp. 75
9. steele richardson olszewski syndrome/ 2129
10. steele-richardson-olszewski disease.tw,mp. 16
11. steele-richardson-olszewski disease/ 2129
12. steele-richardson-olszewski syndrome.tw,mp. 75
13. steele-richardson-olszewski syndrome/ 2129
14. exp steele richardson olszewski syndrome/ 2129
15. PSP-P.tw. 50
16. PSP-CBS.tw. 4
17. PSP-PAGF.tw. 0
18. PSP-RS.tw. 24
19. (progressive supranuclear palsy adj2 parkinsonism).tw,mp. 46
20. Pure akinesia with gait freezing.tw,mp. 11
21. PSP-F.tw. 1
22. PSP-SL.tw. 0
23. PSP-PI.tw. 0
24. PSP-OM.tw. 0
25. PSP-PGF.tw. 0
26. 1 or 2 or 3 or 4 or 5 or 6 or 7 or 8 or 9 or 10 or 11 or 12 3363

or 13 or 14 or 15 or 16 or 17 or 18 or 19 or 20 or 21 or 22

or 23 or 24 or 25

1. exp Prevalence/ or prevalence.mp. 524433
2. prevalence.tw. 446790
3. 27 or 28 524433
4. prevalent.tw. 112671
5. 29 or 30 602348
6. 26 and 31 125
7. Corticobasal Degeneration.tw,mp 1228
8. Corticobasal Degeneration/ 0
9. Corticobasal Syndrome.tw,mp 314
10. Corticobasal Syndrome/ 0
11. 33 or 34 or 35 or 36 1455
12. 37 and 31 52

**31/12/18**

1. exp Supranuclear Palsy, Progressive/ 2267
2. progressive supranuclear pals$.tw,mp. 3175
3. Richardson$ syndrome.tw,mp. 87
4. Richardson's Syndrome/ 2267
5. Richardsons syndrome/ 0
6. steele richardson olszewski disease.tw,mp. 15
7. steele richardson olszewski disease/ 2267
8. steele richardson olszewski syndrome.tw,mp. 75
9. steele richardson olszewski syndrome/ 2267
10. steele-richardson-olszewski disease.tw,mp. 15
11. steele-richardson-olszewski disease/ 2267
12. steele-richardson-olszewski syndrome.tw,mp. 75
13. steele-richardson-olszewski syndrome/ 2267
14. exp steele richardson olszewski syndrome/ 2267
15. PSP-P.tw. 57
16. PSP-CBS.tw. 6
17. PSP-PAGF.tw. 1
18. PSP-RS.tw. 30
19. (progressive supranuclear palsy adj2 parkinsonism).tw,mp. 49
20. Pure akinesia with gait freezing.tw,mp. 16
21. PSP-F.tw. 1
22. PSP-SL.tw. 0
23. PSP-PI.tw. 0
24. PSP-OM.tw. 0
25. PSP-PGF.tw. 0
26. 1 or 2 or 3 or 4 or 5 or 6 or 7 or 8 or 9 or 10 or 11 or 12

or 13 or 14 or 15 or 16 or 17 or 18 or 19 or 20 or 21 or 22

or 23 or 24 or 25 3568

1. exp Prevalence/ or prevalence.mp. 559367
2. prevalence.tw. 478750
3. 27 or 28 559367
4. prevalent.tw. 123296
5. 29 or 30 644499
6. 26 and 31 137
7. Corticobasal Degeneration.tw,mp. 1273
8. Corticobasal Degeneration/ 0
9. Corticobasal Syndrome.tw,mp. 356
10. Corticobasal Syndrome/ 0
11. 33 or 34 or 35 or 36 1536
12. 37 and 31 56
13. limit 38 to yr="2017 - 2019" 6

**1.2 EMBASE**

**03/07/17**

1. progressive supranuclear ophthalmoplegia.tw,mp. 9
2. exp Supranuclear Palsy, Progressive/ 5620
3. progressive supranuclear pals$.tw,mp. 6351
4. Richardson$ syndrome.tw,mp. 197
5. Richardson's Syndrome/ 0
6. Richardsons syndrome/ 0
7. steele richardson olszewski disease.tw,mp. 18
8. steele richardson olszewski disease/ 0
9. steele richardson olszewski syndrome.tw,mp. 121
10. steele richardson olszewski syndrome/ 5620
11. steele-richardson-olszewski disease.tw,mp. 18
12. steele-richardson-olszewski disease/ 0
13. steele-richardson-olszewski syndrome.tw,mp. 121
14. steele-richardson-olszewski syndrome/ 5620
15. exp steele richardson olszewski syndrome/ 5620
16. PSP-P.tw. 148
17. PSP-CBS.tw. 38
18. PSP-PAGF.tw. 9
19. PSP-RS.tw. 86
20. (progressive supranuclear palsy adj2 parkinsonism).tw,mp. 89
21. Pure akinesia with gait freezing.tw,mp. 49
22. PSP-F.tw. 3
23. PSP-SL.tw. 0
24. PSP-PI.tw. 0
25. PSP-OM.tw. 0
26. PSP-PGF.tw. 0
27. exp Prevalence/ or prevalence.mp. 840033
28. prevalence.tw. 689477
29. prevalent.tw. 183158
30. 1 or 2 or 3 or 4 or 5 or 6 or 7 or 8 or 9 or 10 or 11 or 12 or 13 6426 or 14 or 15 or 16 or 17 or 18 or 19 or 20 or 21 or 22 or 23 or 24 or 25 or 26
31. 27 or 28 or 29 962310
32. 30 and 31 326
33. Corticobasal Degeneration.tw,mp 2824
34. Corticobasal Degeneration/ 2246
35. Corticobasal Syndrome.tw,mp 725
36. Corticobasal Syndrome/ 119
37. 33 or 34 or 35 or 36 3188
38. 37 and 31 176

**31/12/18**

1. exp Supranuclear Palsy, Progressive/ 6348
2. progressive supranuclear pals$.tw,mp. 7165
3. Richardson$ syndrome.tw,mp. 244
4. Richardson's Syndrome/ 0
5. Richardsons syndrome/ 0
6. steele richardson olszewski disease.tw,mp. 18
7. steele richardson olszewski disease/ 0
8. steele richardson olszewski syndrome.tw,mp. 123
9. steele richardson olszewski syndrome/ 6348
10. steele-richardson-olszewski disease.tw,mp. 18
11. steele-richardson-olszewski disease/ 0
12. steele-richardson-olszewski syndrome.tw,mp. 123
13. steele-richardson-olszewski syndrome/ 6348
14. exp steele richardson olszewski syndrome/ 6348
15. PSP-P.tw. 182
16. PSP-CBS.tw. 46
17. PSP-PAGF.tw. 12
18. PSP-RS.tw. 115
19. (progressive supranuclear palsy adj2 parkinsonism).tw,mp. 103
20. Pure akinesia with gait freezing.tw,mp. 59
21. PSP-F.tw. 4
22. PSP-SL.tw. 1
23. PSP-PI.tw. 2
24. PSP-OM.tw. 1
25. PSP-PGF.tw. 2
26. 1 or 2 or 3 or 4 or 5 or 6 or 7 or 8 or 9 or 10 or 11 or 12 or 13

or 14 or 15 or 16 or 17 or 18 or 19 or 20 or 21 or 22 or 23 or 24 or 25 7249

1. exp Prevalence/ or prevalence.mp. 948348
2. prevalence.tw. 780978
3. 27 or 28 948348
4. prevalent.tw. 211679
5. 29 or 30 1089994
6. 26 and 31 387
7. Corticobasal Degeneration.tw,mp. 3183
8. Corticobasal Degeneration/ 2572
9. Corticobasal Syndrome.tw,mp. 877
10. Corticobasal Syndrome/ 140
11. 33 or 34 or 35 or 36 3594
12. 37 and 31 211
13. limit 38 to yr="2017 - 2019" 42
14. frontotemporal dementia.mp. or exp Frontotemporal Dementia/ 16752
15. exp Incidence/ 410194
16. exp Prevalence/ or prevalence.mp. 948348
17. 41 or 42 1299147
18. 40 and 43 957
19. limit 44 to yr="2017 -Current" 180
20. progressive supranuclear ophthalmoplegia.tw,mp. 9
21. 26 or 46 7255
22. 47 and 31 388
23. limit 48 to yr="2017 - 2019" 83

**1.3 LILACS**

Keyword searches 18/07/18: “progressive supranuclear palsy” 35 records; “corticobasal degeneration” 12 records.

**1.4 CINAHL**

Keyword searches 18/07/18: “progressive supranuclear palsy” AND “prevalence” 17 records, “corticobasal syndrome” OR “corticobasal degeneration” AND “prevalence” 139 records.

**1.5 Web of Science**

Keyword searches 18/07/18: “progressive supranuclear palsy” AND “prevalence” 278 records; “corticobasal degeneration” AND “prevalence” 97 records.

**2. Studies of prevalence of parkinsonian disorders and Parkinson’s disease**

The electronic search strategies are as follows, including the numbers of results for each search string (for years where systematic review not available):

Ovid Medline and EMBASE 09/11/17:

1. parkinsonism.mp. 51680
2. parkinsonism/ 35535
3. parkinson disease.mp.
4. parkinson disease/ 191083
5. parkinson's disease.mp 166962
6. parkinson's disease/ 182241
7. parkinsonian/ 0
8. parkinsonian.mp. 33118
9. 1 or 2 or 3 or 4 or 5 or 6 or 7 or 8 263126
10. prevalence.mp 1422974
11. prevalence/ 832578
12. prevalent.mp 313086
13. prevalent/ 0
14. 10 or 11 or 12 or 13 1635434
15. 9 and 14 12453
16. limit 15 to yr="2010 - 2013" 3739
17. remove duplicates from 16 2801
18. limit 15 to yr="2014 -Current" 4032
19. remove duplicates from 18 2965
20. limit 15 to yr="1964 - 1985" 177
21. remove duplicates from 20 110

Ovid Medline and EMBASE 31/12/18:

1. parkinsonism.mp. 53711
2. parkinsonism/ 37420
3. parkinson disease.mp. 210320
4. parkinson disease/ 201188
5. parkinson's disease.mp. 176350
6. parkinson's disease/ 192362
7. parkinsonian/ 0
8. parkinsonian.mp. 33512
9. 1 or 2 or 3 or 4 or 5 or 6 or 7 or 8 275586
10. prevalence.mp. 1498240
11. prevalence/ 887764
12. prevalent.mp. 335015
13. prevalent/ 0
14. 10 or 11 or 12 or 13 1725445
15. 9 and 14 13520
16. limit 15 to yr="2017 - 2018" 2030
17. remove duplicates from 16 1683
18. limit 15 to yr="2018" 899
19. remove duplicates from 18 829

**3. Studies of incidence or prevalence of frontotemporal dementia**

**3.1 Ovid Medline 06/11/17**

1. frontotemporal dementia.mp. or exp Frontotemporal Dementia/ 5705
2. exp Incidence/ 240245
3. exp Prevalence/ or prevalence.mp. 559294
4. 2 or 3 764041
5. 1 and 4 273

**3.2 Embase 10/11/17**

1. frontotemporal dementia.mp. or exp Frontotemporal Dementia/ 15283
2. exp Incidence/ 359997
3. exp Prevalence/ or prevalence.mp. 873051
4. 2 or 3 1180136
5. 1 and 4 850

**4. Systematic Reviews of incidence and prevalence of PSP, CBD, parkinsonism or FTD**

1. Progressive supranuclear palsy.mp 9906
2. corticobasal degeneration.mp 4259
3. incidence.mp 1815240
4. prevalence.mp 1434633
5. 1 or 2 11584
6. 3 or 4 3065600
7. systematic review.mp 293165
8. meta analysis.mp. 340882
9. 7 or 8 503727
10. 5 and 6 and 9 16
11. parkinsonism.mp. 51954
12. parkinson disease.mp. 201295
13. 11 or 12 230047
14. 6 and 9 and 13 617
15. 6 and 9 and 12 517
16. frontotemporal dementia.mp 19359
17. 6 and 9 and 16 74
18. remove duplicates from 17 47

**1/11/18**

1. review.ab 958487
2. review.pt. 2290757
3. meta-analysis.ab. 77977
4. meta-analysis.pt. 93492
5. meta-analysis.ti. 62407
6. or/1-5 2632233
7. letter.pt. 948763
8. comment.pt. 690548
9. editorial.pt. 420277
10. or/7-9 1525929
11. 6 not 10 2595426
12. Progressive supranuclear palsy.mp. 3153
13. corticobasal degeneration.mp. 1266
14. corticobasal syndrome.mp. 352
15. incidence.mp. 694327
16. prevalence.mp. 554117
17. 12 or 13 or 14 3845
18. 15 or 16 1178278
19. 11 and 17 and 18 38
20. parkinsonism.mp. 14931
21. parkinson disease.mp. 65933
22. 20 or 21 72538
23. frontotemporal dementia.mp. 5092
24. 11 and 22 and 18 1033
25. 11 and 23 and 18 85
26. meta-analy:.mp. 242869
27. search:.tw. 493744
28. review.pt. 2412971
29. or/26-28 2876655
30. 29 and 17 and 18 117
31. 29 and 22 and 18 2329
32. 11 and 23 and 18 294
